# A multi-center survey on hospital malnutrition and cachexia in Slovenia

**DOI:** 10.1038/s41430-019-0485-y

**Published:** 2019-08-06

**Authors:** Barbara Koroušić Seljak, Denis Mlakar Mastnak, Živa Mrevlje, Gregor Veninšek, Nada Rotovnik Kozjek

**Affiliations:** 1 Computer Systems, Jožef Stefan Institute, Jamova c. 39, Ljubljana, Slovenia; 2 Institute of Oncology Ljubljana, Zaloška c. 2, Ljubljana, Slovenia; 3 King’s College Hospital NHS Foundation Trust, Institute of Liver Studies, London, UK; 4 Peter Držaj Hospital, University Medical Centre Ljubljana, Vodnikova 62, Ljubljana, Slovenia; 5 Medical Faculty, University of Ljubljana, Vrazov trg 20, Ljubljana, Slovenia

**Keywords:** Risk factors, Nutrition

## Abstract

**Background:**

Malnutrition has become a prevalent condition, with European and international studies reporting rates of approximately 25–40% in hospitals. We set out to perform a multi-center cross-sectional study to assess malnutrition rates in Slovenian hospitals and to convert the findings into a mobile application suitable for use by nurses and staff at the bedside. In addition, we examined the association of the results of this mobile application with parameters for body composition measured by bioimpedance method, muscle strength, anthropometrics, and specific blood markers.

**Methods:**

We selected the Nutritional Risk Screening 2002 (NRS-2002) method, the second version of the modified short-form of Mini-Nutritional Assessment (MNA-SF), and the diagnostic criteria for cachexia proposed by Evans (CDE) as evidence-based methods for estimating the risk of and prevalence of malnutrition or/and cachexia. The methods were converted into the Android mobile application named MalNut that was used in three Slovenian hospitals by nurses and dietitians.

**Results:**

We applied NRS-2002 and MNA-SF to screen for malnutrition risk and to assess malnutrition in 207 individuals aged 18 years and older, regardless of gender or reason for hospitalization during 1-week periods. Totally, 98% of these patients consider nutrition an important part of medical treatment care. NRS-2002 estimated the malnutrition risk to be 66.3%, which includes both patients to be at risk for malnutrition and patients that are truly malnourished. The malnutrition risk in the elderly (65+) estimated by MNA-SF was 39.6% and malnutrition 42.5%. When applying the CDE score in these two categories, 66.7% were identified as cachectic and 21.4% as pre-cachectic. In the patients assessed with the CDE score, malnutrition risk increased with higher extracellular water and decreased body mass index, hemoglobin, phase angle, and muscle strength. In all, 75% of patients assessed as high risk for malnutrition by NRS-2002, were identified as cachectic and 15.7% as pre-cachectic. In NRS-2002 assessed patients, this risk increased with higher C-reactive protein and lower phase angle.

**Conclusions:**

The study showed that both malnutrition and cachexia are largely overlapping notions and are common in hospitalized adults in Slovenia. The MNA-SF and NRS-2002 tools showed that malnutrition risk was not significantly correlated with age, gender, serum albumin, but was correlated with lower phase angle, CRP, and muscle strength in elderly patients. The results have been used to develop further nutritional interventions in Slovenia.

## Introduction

Malnutrition is common worldwide phenomenon and represents a major medical and economical issue, also in developed countries. However, it is difficult to assess the magnitude of the problem as malnutrition represents a wide variety of ailments like exclusive lack of sufficient food intake, leading to starvation related loss of fat mass and fat-free mass, and inflammation related sarcopenia and cachexia in inflammatory conditions, like disease, trauma, cancer, and ageing. All these conditions are frequently encountered in daily practice [1–4]. As differentiation between nutrition-related disorders is still infrequently performed in clinical practice [5], there is an urgent need in Slovenia to increase awareness in health care regarding nutrition-related health problems as unrecognized starvation, sarcopenia and cachexia, and propose appropriate methods for identification and treatment of each of these conditions.

### Malnutrition

Malnutrition means poor nutrition and encompasses both undernutrition and overnutrition. In the case of chronic disease and its impact on dietary intake and nutritional status, the term malnutrition generally describes “a disordered state of nutrition, caused by a varying combination of a negative (or positive) nutrient balance and inflammation, leading to abnormal body composition and function [1, 6, 7]. Importantly, many overweight people with a high body mass index (BMI) suffer from insufficient nutritional intake, weight loss, and poor intake.

The prevalence of malnutrition is high. European studies and studies outside Europe report rates of approximately 25–40% in hospitals, 20–25% in nursing homes, and 15–20% at home [8]. When nutritional requirements are not met, the impact on both malnourished patient and society can be high. In Europe, the costs associated with managing the consequences of malnutrition were estimated to amount to at least 170 billion euros [9, 10].

Optimizing nutritional intake is simple and relatively inexpensive, especially when detected early. Yet, many chronically ill and older patients are at risk to eat insufficient amounts of high-quality food due to the low priority given to ensuring adequate nutritional care for all patients, whether living at home, in the hospital or in long-term care centers. However, the inflammatory activity induced by chronic disease and to some degree also by the ageing process itself is difficult to counter. This inevitably leads to gradual muscle loss. However, adequate nutrition remains important, to counter additional ill-effects of inadequate nutritional intake.

### Cachexia

Cachexia is a very similar notion as malnutrition and is a multifactorial and complex metabolic syndrome associated with underlying illness and defined by an ongoing loss of skeletal muscle with or without concomitant loss of fat, which cannot be fully reversed by conventional nutritional support and leads to progressive functional impairment [1]. Its pathophysiology is characterized by a negative energy and protein balance due to a variable combination of reduced food intake and metabolic changes [11].

Several chronic diseases, such as cardiovascular disease, cancer, and chronic respiratory diseases (COPD), are conditions that contribute to cachexia. Cachexia prevalence in these disease states amounts to 28–57% in cancer, 16–42% in chronic heart failure, and 27–35% in COPD [12]. Moreover, a recent estimate suggests that approximately nine million patients suffer from cachexia due to chronic disease [13]. However, to tackle the medical and economical problem of cachexia in different countries and various clinical settings, we have to identify the cachexia magnitude in our local environment.

### Situation in Slovenia

In Slovenia, the national nutritional recommendations for malnutrition screening were published in 2008 [14]. The Slovenian multidisciplinary consensus on the international definition, staging, clinical classification, and multimodal approach to cachexia treatment in cancer patients was adopted in 2011 [15]. However, in the majority of hospitals and clinical settings, a multidisciplinary approach to malnutrition and cachexia is still a missing link in medical evaluation.

The purpose of the Slovenian multi-center and cross-sectional study presented in this paper was to utilize evidence-based methods to estimate the prevalence of malnutrition and cachexia in hospitalized adult patients. The additional objective was to examine the association between demographic, anthropometric, certain biochemical, and body composition parameters with impaired nutritional status assessed with the mobile bedside application. The secondary aim was to validate the method and use the data obtained as a platform to develop further nutritional interventions.

## Methods

**Fig. 1:**
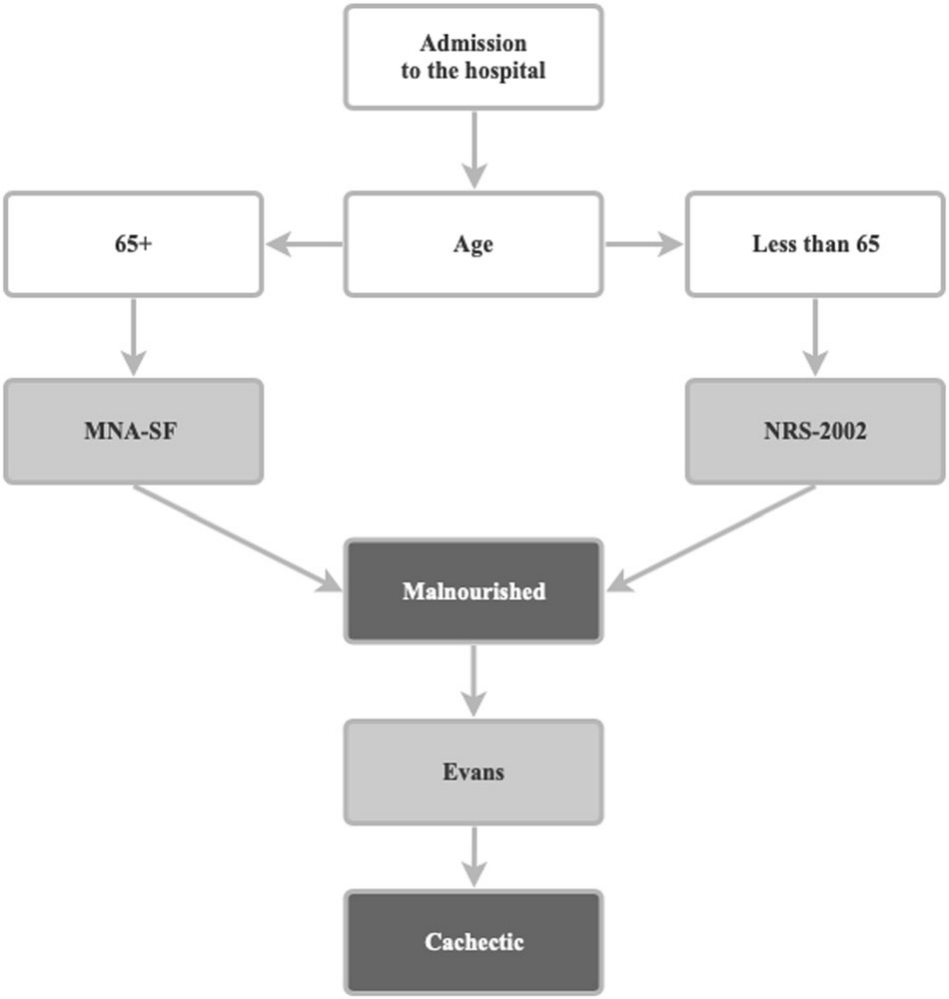
Malnutrition and cachexia screening tools supported by the mobile application MalNut

We selected the following evidence-based methods for estimating the prevalence of the risk malnutrition by two standard scoring tools and according to the consensus definition of cachexia (see Fig. 1):

- *Nutritional Risk Screening 2002 (NRS-2002)*: NRS-2002 is a tool for nutritional risk screening based on the analysis of 128 trials. It is based on the concept that nutritional support is required in patients who are severely ill with increased nutritional requirements, or who are severely undernourished, or who have certain degrees of severity of disease in combination with certain degrees of undernutrition [16]. This tool has been recommended by ESPEN for clinical settings.
- *The Mini-Nutritional Assessment (MNA)*: The MNA is a screening tool developed for detecting the presence of malnutrition risk in the elderly. Originally developed as a 30-point measurement, the MNA has been modified into a short form (MNA-SF) to provide a more practical tool while preserving the accuracy of the original. The classical MNA-SF comprises six questions addressing food intake and appetite, weight loss, mobility, acute illness or stress, dementia or depression, and BMI. The classical MNA-SF offers a rapid tool for excluding well-nourished older people, but does not differentiate between the nutritionally at risk and the malnourished individuals as in the original MNA. Therefore, we used the new MNA-SF which enables the replacement of BMI with calf circumference when BMI is not available and can clearly differentiate among the three categories of well-nourished, at risk for malnutrition, and malnourished patients. The measurement is easy to implement in clinical settings and takes less than 4 min to complete. In 2009, the MNA-SF was revalidated in a receiver operating characteristics analysis of 12 databases involving 2032 study participants from around the world [17]. Results showed that the new MNA-SF score correlates strongly with the full MNA score in this global data set. The new MNA-SF has been recommended by ESPEN for older patient.
- *Cachexia diagnosis proposed by Evans (CDE)*: This diagnosis is based upon a definition that cachexia results from the adaptation to an underlying illness such as cancer [1]. The illness creates an environment that may be characterized by inflammation, loss of appetite (anorexia), low levels of testosterone and other anabolic hormones, and anemia. Decreased food intake and anorexia result in loss of body and muscle mass. In addition, inflammation, insulin resistance, and low levels of anabolic hormones result in muscle wasting. Evidence-based results exist [18], showing a substantial difference in the prediction of overall survival when comparing the diagnostic guidelines according to Fearon et al. [11] with the diagnostic guidelines according to Evans et al. [1].

The evidence-based methods NRS-2002, MNA, MNA-SF, and CDE were converted into a mobile application (named MalNut) that can be used across all health and care settings by nurses and dietitans [19]. The application is easy to use and can run on an Android powered smartphone or tablet. As it provides decision-making questions upon each step, it allows rapid assessment.

In addition to the nutritional and screening tools, we added a general question about the patient’s awareness of the importance of nutritional treatment during the illness.

### Study design

This multi-center and cross-sectional study utilized NRS-2002, MNA, MNA-SF, and CDE tools to screen and assess all newly admitted patients hospitalized at the following Slovenian hospitals: the Oncology Institute Ljubljana (OI), the Gastroenterology Clinic of the Medical Center Ljubljana (GC), and the Peter Držaj (PD) Hospital Ljubljana. Screening and assessment were conducted for the risk of malnutrition and cachexia.

The algorithm design is presented in Fig. 1. For study participants of age 65+, the MNA-SF was utilized, whereas for other adults, NRS-2002 was utilized. In both cases, if the study participant had been assessed as malnourished or at risk for malnutrition, CDE was conducted to assess cachexia.

For MNA-SF, a total score of ≤7 indicated malnutrition and a total score of 8–11 a risk for malnutrition, while for NRS-2002 a total score of ≥3 indicated an increased risk for malnutrition, also including malnutrition. For CDE, the malnourished patients were diagnosed with cachexia if they were facing at least three out of five health problems (decreased muscle strength, fatigue, anorexia, low fat-free mass index, or abnormal biochemistry—CRP > 5 mg/l, Hb < 12 g/dl, SA < 3.2 g/dl) and losing at least 5% of weight in 12 months or less (or having BMI < 20 kg/m^2^).

The present study was approved by the Slovenian Medical Ethics Committee as an observational (no intervention) study.

### Study population

The study group consisted of a convenience sample of 207 individuals aged 18 and older, regardless of gender or reason for hospitalization, admitted to the OI, GC, and PD hospitals during 1-week periods from June to September 2012. In OI, GC, and PD, respectively 150, 42, and 15 subjects admitted to all inpatient departments were included.

All adult patients newly admitted to the hospitals were eligible for participation in the present study. Excluded individuals were those who expired prior to undergoing the nutrition screen, and a few patients, who were too weak to participate. All study participants signed a written consent form. During the study no patient withdrew the consent. Before signing the informed consent, we asked patients “what they think about nutrition” in order to assess their awareness of the importance of nutrition care during illness and disease treatment.

### Demographics and laboratory values

Demographic characteristics, including participant age, gender, and community health center were extracted either from the patient’s medical record or elicited from the patient if not recorded in the medical record. Blood values determined on admission, including hemoglobin concentration (Hb), C-reactive protein (CRP), and serum albumin (SA), were extracted from the patient record. Both demographics and blood values were entered into the mobile application MalNut to be considered in the screening procedure.

### Anthropometric measures

The study participants were weighed on a hospital scale in the room in which they were hospitalized. For patients, who could not stand for weighing, weight was self-reported. The patient himself/herself reported the height. Self-reported weight and height have been reported to be acceptable substitutes for directly measured weight and height when using the NRS-2002 [16].

To reduce variability, weight and height were performed by well-trained dietitians who were in charge of data acquisition. The data were entered into the mobile application MalNut, which automatically calculated BMI as weight (kg)/height (m^2^) using either directly measured or estimated values for weight and height.

### BIA and muscle strength measures

Body composition of the study participants was estimated by bioelectrical impedance analysis (BIA), while muscle strength was measured by a hand dynamometer. Both measurements were performed by well-trained dietitians in the rooms in which the study participants were hospitalized, and stored in MalNut. Patients were in the supine position while body composition was measured. Muscle strength was measured in the upright position, as recommended. In few bedridden patients, it was measured in the lying position.

Bioelectrical impedance was measured by the BodyStat’s QuadScan 4000 using several frequencies (50, 100, 150, 200 kHz). At these frequencies, BIA was able to predict body fat, lean body mass, total body water, fat-free body mass (FFM), extracellular water (ECW), intracellular water, body fat mass index, FFM index, and phase angle (PA).

The patient’s muscle strength was measured by the Baseline hydraulic hand dynamometer 12-0240.

### Data quality assurance

All documentation relating to the survey has been open to inspection.

### Statistical analysis

A convenience sample of 207 individuals hospitalized at the OI (150 patients), GC (42 patients), and PD (15 patients) participated in the study during the data acquisition period. In the study design, a sample size of 600 individuals was anticipated, which would provide a confidence level of 95% and a 4% margin of error to estimate the prevalence of malnutrition. Because of the study time constraint, only 207 subjects were sampled during the data acquisition period. This sample size provided a prevalence estimate confidence level of 95% and a 7% margin of error.

Data were recorded by the mobile application MalNut that is connected to the Open Platform for Clinical Nutrition (OPEN, http://opkp.si) for data storage and analysis. The analysis results provided by OPEN were imported into SPSS v. 21.0 (SPSS Inc., Chicago, IL, USA), which was used for statistical analysis.

Distributions of continuous variables were assessed for normality using the Shapiro–Wilk test (cutoff *P* < 0.01) that is more appropriate for small sample sizes (<50 samples), but can also handle sample sizes as large as 2000. If the Sig. value of the Shapiro–Wilk test was greater than 0.05, the data were treated as normal.

The proportion of malnourished individuals was estimated by dividing the number of malnourished individuals by the total number of the surveyed patients. Continuous and categorical variables were described using mean ± standard deviation and by frequency counts expressed as *n* (%), respectively. Continuous variables were compared with high malnutrition risk using the unrelated *t* test or the Mann–Whitney *U* test for independent measures.

Associations between continuous variables were described by calculating Pearson’s or Spearman’s correlation coefficient as appropriate, while associations between categorical variables were assessed using the chi-square test. All the tests were two-sided and considered as significant at *P* < 0.05.

## Results

A total of 207 adults (108 females and 99 males) participated in the study. This represented 73% of all individuals admitted to the participating hospitals during the data acquisition period.

Totally, 98% of patients considered nutritional care to be an important treatment measure.

The NRS-2002 was utilized for 101 adult study participants of age less than 65 years. Altogether, 67 patients (66.3%) were identified as at risk for malnutrition (Score of the final screening ≥3). The response patterns to the NRS-2002 is shown by malnutrition risk status in Table 1. Among 67 patients at risk of malnutrition, 51 (76.0%) patients were identified as cachectic, while 8 of these patients suffered from cancer cachexia.

**Table 1:** Distribution of NRS-2002 scores by malnutrition risk status

	Malnutrition risk not elevated (*n* = 34)	malnutrition risk (*n* = 67)	*P* value
*Section 1 (% answering “yes”)*			
Is BMI < 20.5	0.0	20.7	0.00
Has the participant lost weight within the last 3 months?	0.0	31.1	0.00
Was the patient’s dietary intake less than usual during the past week?	68.9	70.1	< 0.01
Is the patient severely ill? (e.g., in intensive therapy)	76.5	88.1	0.98
*Section 2 (score)*			
Impaired nutritional status score	1.1 ± 0.2	1.7 ± 1.3	0.00
Severity of disease score	0.9 ± 0.3	1.3 ± 0.5	0.00
Total NRS 2002 Score	1.4 ± 0.6	3.7 ± 0.6	0.00
*Cachexia (number)*	0	51	0.00
Cancer cachexia	0	8	0.00

The MNA-SF was utilized for 106 adult study participants of age 65 years and older. Among these participants, 42 patients (39.6%) were identified as malnourished (score of the final screening ≤7) and 45 (42.5%) at risk of malnutrition. This reflects impaired nutritional status in 76.1% of elderly. The response patterns to the MNA-SF is shown by malnutrition risk status in Table 2. Among 42 malnourished patients, 28 (66.7%) patients were identified as cachectic.

**Table 2:** Distribution of MNA-SF scores by malnutrition risk status

	Normal nutrition status (*n* = 34)	Malnourished (*n* = 42)	*P* value
*Section 1 (score)*			
Total MNA-SF Score	10.4 ± 1.5	4.7 ± 1.6	0.00
*Section 2 (score)*			
Cachexia (number)	0	28	0.00
Cancer cachexia	0	6	0.00

The NRS-2002 and MNA-SF populations are listed according to nutritional status in Table 3. It is shown that in NRS-2002 assessed patients the malnutrition risk did not differ with age, gender, BMI, hemoglobin, SA, and most BIA parameters. Subjects at high malnutrition risk had significantly higher disease marker CRP (30.4 ± 58.0 vs. 6.6 ± 10.1), *t*(59) = 2.9, *P* < 0.01, and lower PA ((4.8 ± 1.4 vs. 5.7 ± 1.0), *t*(98) = −3.3, *P* < 0.01). Both CRP and PA were weakly correlated with total NRS scores. While the correlation between CRP and total NRS scores was positive but statistically insignificant (rho (101) = 0.2, *P* = 0.08), the correlation between the PA and total NRS scores was negative and statistically significant (rho (101) = −0.3, *P* < 0.01).

**Table 3:** Subject characteristics by malnutrition risk status

NRS-2002				MNA-SF		
	Malnutrition risk not elevated (*n* = 34)	Malnutrition risk (*n* = 67)	*P* value	Normal nutrition status (*n* = 24)	Malnourished (*n* = 42)	*P* value
Age (years)	49.2 ± 11.3	50.3 ± 10.7	0.65	72.5 ± 6.8	72.8 ± 8.1	0.88
Females (%)	49.3%	52.9%	0.73	51.6%	57.1%	0.58
BMI (kg/m^2^)	26.5 ± 3.4	25.5 ± 6.3	0.33	27.5 ± 4.4	23.3 ± 5.5	0.00
*Complete blood count*						
K-Hb	128.5 ± 19.3	121.8 ± 21.9	0.15	124.3 ± 15.7	117.0 ± 15.	0.03
*Disease marker*						
CRP	6.6 ± 10.1	30.4 ± 58.0	< 0.01	13.4 ± 27.1	27.0 ± 40.6	0.21
*Nutrition marker*						
Serum albumin	31.9 ± 19.2	38.3 ± 7.3	0.33	33.0 ± 15.3	34.7 ± 6.4	0.05
*BIA*						
FAT	27.9 ± 8.9	27.7 ± 10.5	0.94	33.7 ± 8.7	33.0 ± 10.2	0.72
LEAN	72.1 ± 9.0	72.2 ± 10.5	0.98	66.3 ± 8.7	67.0 ± 10.2	0.72
TBW	52.6 ± 7.2	53.8 ± 12.6	0.56	53.7 ± 6.9	56.3 ± 12.4	0.16
ECW	28.9 ± 3.6	25.9 ± 8.5	0.09	23.3 ± 4.7	26.6 ± 7.2	0.01
ICW	29.4 ± 3.4	28.8 ± 6.4	0.90	28.4 ± 3.8	28.8 ± 4.7	0.69
BFMI	7.4 ± 2.8	7.1 ± 4.6	0.71	9.1 ± 3.5	7.6 ± 3.9	0.04
FFMI	16.7 ± 6.0	17.5 ± 4.1	0.45	17.9 ± 3.4	16.1 ± 3.1	0.42
Phase angle	5.7 ± 1.0	4.8 ± 1.4	<0.01	4.6 ± 1.0	3.6 ± 1.1	0.00
Muscle strength	39.8 ± 14.7	35.5 ± 13.5	0.98	30.7 ± 10.0	23.7 ± 9.3	<0.01

In MNA-SF assessed patients, malnourished subjects had significantly lower BMI (23.3 ± 5.5 vs. 27.5 ± 4.4, *P* = 0.00), significantly lower K-Hb (117.0 ± 15 vs. 124.3 ± 15.7, *P* = 0.03), significantly higher ECW (26.6 ± 7.2 vs. 23.3 ± 4.7, *P* = 0.01), significantly lower PA (3.6 ± 1.1 vs. 4.6 ± 1.0, *P* = 0.00), and significantly lower muscle strength (23.7 ± 9.3 vs. 30.7 ± 10.0, *P* < 0.01). The correlation between BMI and total MNA-SF scores was positive and statistically significant (rho(106) = 0.5, *P* = 0.00). Also the correlation between K-Hb and total MNA-SF scores was positive and statistically significant (rho(106) = 0.2, *P* = 0.03), while the correlation between ECW and total MNA-SF scores was negative and statistically significant (rho(106) = −0.3, *P* = 0.01). The correlation between PA and total MNA-SF scores was positive and statistically significant (rho(106) = 0.4, *P* = 0.00), as well as the correlation between muscle strength and total MNA-SF scores (rho(106) = 0.3, *P* < 0.01).

**Fig. 2:**
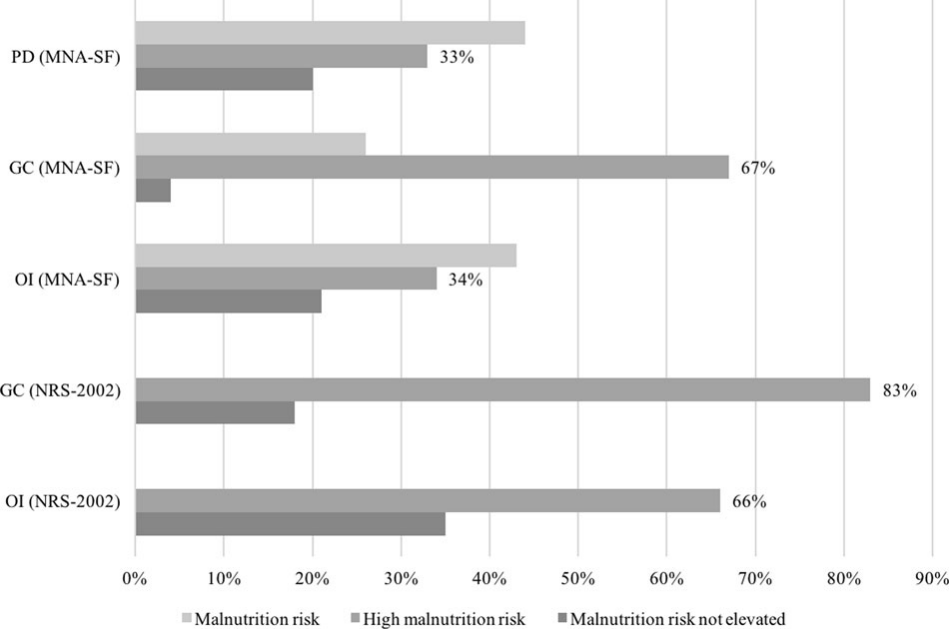
Malnutrition state in different clinical settings

Figure 2 shows malnutrition risk by clinical settings/illness in our sample. The highest risk of malnutrition was observed at the GC (at high malnutrition risk were 67% of older hospitalized patients (aged 65+) and 83% of other hospitalized adults). Comparing the study results for different hospital settings, we concluded that the situation with malnourished patients was extremely bad at the GC; 83% of NRS-2002 patients and 67% of MNA-SF patients were diagnosed as malnourished (Fig. 2). The percentage of malnourished older patients (aged 65+) (33%) in the PD was comparable with the percentage of malnourished cancer patients (34%) in the OI. However, the group of patients from PD was relatively small (15 patients).

## Discussion

The multi-center and cross-sectional study on malnutrition and cachexia presented in this paper showed that both conditions are common among hospitalized adults in Slovenia. As only four (2%) participants answered that they do not believe in the importance of nutritional care, we can conclude that awareness among patients is high, supporting activities to improve nutritional state that is often prevailing in health care.

As there is no international consensus on a single “best” nutrition screening and assessment tool, we applied the MNA-SF and NRS-2002 tools to screen and assess older patients (aged 65+) and other adults, respectively. While the malnutrition risk in the elderly estimated by MNA-SF was 39.6%, the NRS-2002 estimated the malnutrition risk to be 66.3%. However, as NRS 2002 screens risk for malnutrition, part of these patients is malnourished already. Both tools showed that malnutrition risk was unrelated to age, gender, SA, and most BIA parameters. In MNA-SF assessed patients, the malnutrition risk increased with higher ECW and lower BMI, Hb, PA, and muscle strength. In NRS-2002 assessed patients, this risk increased with higher CRP and lower PA. Our results for MNA-SF assessing the older patients are consistent with reports from other hospitalized populations in Switzerland, China, Italy, and Israel [20–23]. However, the comparison of our results for NRS-2002 patients, indicated that our survey recorded a much higher prevalence of nutritional risk relative to that observed in the Swiss, Chinese, Italian, and Israel surveys. While the prevalence observed in these studies ranged from 20% in Switzerland [20] to 32% in Israel [23], our study showed that approximately two in three hospitalized adults less than 65 years of age were at risk for malnutrition.

Among MNA-SF patients assessed as malnourished, 66.7% patients were identified as cachectic. Among NRS-2002 patients assessed as high risk for malnutrition, 76.0% patients were identified as cachectic. Patients with cachexia usually have a low SA and CRP values slightly above normal. Increasing levels of CRP provide a rough measure of chronic inflammation. In a weight-losing patient with a normal SA and a normal or slightly elevated CRP, the physician should be particularly alert for alternate causes for weight loss. Our survey showed for both MNA-SF and NRS-2002 assessed patients, there was a statistically significant correlation between cachexia and CRP (*P* < 0.01), while the correlation between cachexia and SA was not statistically significant. SA was measured in a selected group (65.1%) of participants.

Our data also indicate that body composition measurement with bioimpedance is a useful clinical tool. We confirmed the value of PA as an additional clinical marker for nutritional risk detection, what was already shown by ref. [24].

Results also reflect differences in malnutrition and cachexia prevalence among hospitals. Despite the IO being a specialized cancer hospital, the prevalence of both nutritional disorders is lower, possibly because this hospital has established a clinical nutrition unit (Fig. 2).

## Conclusion

Malnutrition and cachexia are prevalent nutritional disorders in selected Slovenian hospitals. As it represents an important medical problem for patients and the health system, there is an urgent need to address the problem more systematically. The data presented here, showed that organized nutritional care provides a good starting point. Moreover, the mobile application MalNut, which combines various evidence-based methods for diagnosing malnutrition and cachexia, simplifies the assessment process of the degree of malnutrition and cachexia in hospitalized patients and the elderly. The application offers possibility for further differentiation between malnutrition risks and different forms of manifest malnutrition. In addition, it can be easily applied in other clinical settings and elderly homes to support the first step toward developing a comprehensive nutritional care protocol within the framework of the hospital accreditation process. Clearly, a larger study involving a larger group of different patients as well as more hospitals and elderly homes are needed to confirm the outcome of this survey in Slovenia.
